# Evidence of Improvement of Lower Limb Functioning Using Hydrotherapy on Spinal Cord Injury Patients

**DOI:** 10.3390/biomedicines11020302

**Published:** 2023-01-21

**Authors:** Liliana Elena Stanciu, Madalina Gabriela Iliescu, Liliana Vlădăreanu, Alexandra Ecaterina Ciota, Elena-Valentina Ionescu, Claudia Ileana Mihailov

**Affiliations:** 1 Department of Physical Medicine and Rehabilitation, Faculty of Medicine, “Ovidius” University of Constanta, 1 University Alley, Campus–Corp B, 900470 Constanta, Romania; 2 Department of Reumatology, Faculty of Medicine, “Ovidius” University of Constanta, 1 University Alley, Campus–Corp B, 900470 Constanta, Romania

**Keywords:** aqua therapy, hydrotherapy, human, rehabilitation treatment, spasticity, spinal cord injury

## Abstract

Background: Spinal cord injury (SCI) is a devastating problem for modern society, whether it affects young people in the most productive period of their lives or the elderly. The spinal cord injury is currently without curative treatment and the therapeutic intervention aims to minimize secondary complications and maximize residual function through rehabilitation medicine. The main objective of this scientific paper is to determine whether there is evidence in the literature regarding the importance and/or use of hydrotherapy, as part of the therapeutic management of the SCI patient, in order to decrease the degree of spasticity, of pain symptoms, increase or maintain range of motion, improve respiratory, cardiovascular, and metabolic status, as well as improve function and psychological benefits. Methods: Using Preferred Reporting Items for Systematic Reviews and Meta-Analyses (PRISMA) procedures, the following databases were analyzed between 2000 and 2021: Pub Med, Pub Med Central, Science Direct, Scopus, and SpringerLink. Initial keywords: rehabilitation treatment, spinal cord injury. Additional keywords: hydrotherapy, aqua therapy, spasticity, human. For the scientific quality of the included articles, risk of bias was assessed using the Downs and Black Appraisal Modified Scale. Results: Our research used only four publications as per PRISMA protocol, assessed with Downs and Black Scale. The study models used in the individual studies included in the research are the following: two systematic reviews, one experimental non-randomized control, and one individual semi-structured interview. Due to the low number of studies, despite two of them being reviews, there is the necessity for a more standardized methodology to prove the benefits hydrotherapy for SCI patients for the improvement of lower limb functioning. Conclusion: Hydrotherapy is an important component of the treatment of an SCI patient, despite the limited number of scientific studies that support this aspect. Clinical trials in the future are required.

## 1. Introduction

Spinal cord injury is a serious pathology that can cause various aspects of a patient’s life to significantly deteriorate. The primary goal of rehabilitation is to improve a patient’s functional level and decrease their secondary morbidity. In this paper, we review various secondary long-term complications that can occur after a spinal cord injury (SCI). Some of these include respiratory, cardiovascular, urinary, and intestinal difficulties, as well as spasticity, pain syndromes, pressure ulcers, and fractures [1].

Most of the time, treating patients with spasticity using drugs can lead to side effects. Hydrotherapy can assist in reducing the amount of medication needed as part of rehabilitation treatment [2].

Spasticity is a major issue for SCI patients, also limiting a patient’s mobility and affecting their ability to perform various activities of daily life. Spasticity represents a significant challenge, both for the patient and for the rehabilitation team [3].

Medical hydrotherapy has been prescribed since Hippocrates revealed the curative virtues of water hundreds of years ago [4].

During the 1800s, Sebastian Kneipp, the ‘founder of hydrotherapy’, wrote extensively about the healing effects of water. His research was immediately recognized by healthcare professionals [5].

Hydrotherapy can relieve pain for patients with various conditions [6]. It can also improve their sensory perception by blocking the nociception signals [7,8]. Additionally, warm water can help nourish the body and reduce the effects of lactic acid and other chemicals in the body. It can also decrease the pain threshold and improve muscle relaxation [9,10].

Giesecke defined various aquatic exercise goals, including decreased spasticity, improved respiratory status, increased endurance, and psychological benefits. The temperature of the water is critical because excessively hot or cold temperatures may exacerbate spasticity and can also decrease the effectiveness of certain therapeutic techniques [11]. Warm waters allow individuals with a spinal cord injury to move freely in ways that would be unpleasant and difficult to do on land, and also help avoid the fear of falling. As a result, many physical and functional aims can be accomplished easier [11].

Unfortunately, aquatic therapy is not routinely available to patients with a spinal cord injury. Many of them are vulnerable to colostomies and frequent involuntary incontinence due to neurogenic bowel dysfunction [12].

According to Recio and Cabahug, aqua-based therapy could be effective for treating spinal cord-injured patients with suprapubic and Foley indwelling catheters, tracheostomy tubes, and pressure ulcers [13]. The patients exhibited no signs of dehydration or respiratory problems during the hydrotherapy sessions. Additionally, in patients with suprapubic and indwelling catheters, there was no evidence of a catheter being pulled [13,14,15].

The primary goal of this study is to determine the importance of hydrotherapy as a part of the therapeutic management of SCI patients on a global scale and whether there is up-to-date literature to confirm our clinical findings.

## 2. Methods

To conduct the research, the following databases: PubMed, PubMed Central, Science Direct, Scopus, and SpringerLink were analyzed between 2000 and 2021, according to the PRISMA procedures recommendations for systematic reviews [16]. Initial keywords—rehabilitation treatment, spinal cord injury. Additional keywords—hydrotherapy, aqua therapy, spasticity, human. Downs and Black Appraisal Modified Scale was used to evaluate the scientific quality of the included articles.

Titles and abstracts were read and evaluated for inclusion using the following criteria:

(1) Population (patients with neuromotor or neuromuscular deficits due to spinal cord injury);
(2) Intervention (hydrotherapy/aqua therapy).

Exclusion criteria included publications from previous time periods, publications about hydrotherapy that have as an aim other pathologies than spinal cord injury and publications that do not use English as the publishing language.

The scientifical quality of the studies was evaluated through the modified Downs and Black Scale [17,18], which is a validated and reliable method for assessing the effectiveness of randomized controlled and noncontrolled trials. The modified Downs and Black Scale has 27 items that are focused on five broad categories, namely reporting (10 items), internal validity—bias (7 items), internal validity—confounding (6 items), power (1 item), and external validity. The scale’s overall score goes from 0 to 28. A study of superior quality has a higher value of the maximum possible score. The overall quality of the papers was: 80% (very good), 70–79% (good), 50–69% (fair), <50% (weak) (Downs and Black 1998).

For possible addition, one author examined titles and abstracts. Another two separately identified the full text of collected articles, with differences addressed by consensus or arbitration from a third author.

The study initially started with a search in the databases mentioned above for a shorter period, namely 2015–2021. Given the small number of articles eligible for a review to demonstrate the importance of hydrotherapy in the therapeutic management of patients with spinal cord injury, the search period was extended to 2000–2021, keeping the same databases. Unfortunately, the number of items identified as eligible did not increase significantly.

The International Committee of Medical Journal Editors suggested, in 1993, that articles submitted to biomedical journals should follow the same set of guidelines.

We chose articles published in 2000 and after because we supported that during that period the International Committee of Medical Journal Editors’ suggestion had been embraced by relevant researchers and had increased the quality of reports [19].

## 3. Results

**Figure 1:**
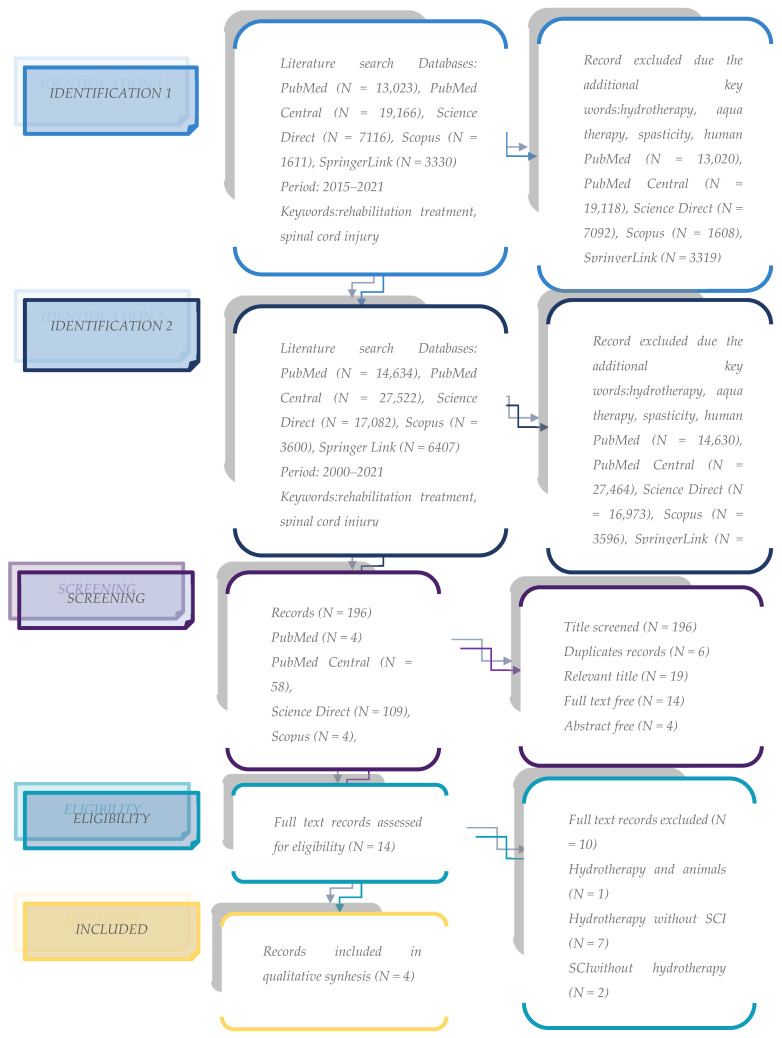
Flow chart of the review process. N—number of records; SCI—spinal cord injury.

As per Figure 1 and Table 1, the algorithm used in finding and selecting the articles shows that only four remained for the final analysis. The years of publication for the articles were 2004, 2017, 2018, and 2019. The main characteristics of the articles are described in Table 2. For further information, please refer to the bibliography of the mentioned studies.

The first study selected was a study published in 2004 by Kesictas N. et al., where researchers evaluated the effectiveness of aquatic therapy on patients with spinal cord injuries. Twenty-four participants were divided into two groups. The first group received standard aquatic exercise twice a day and the other group received oral baclofen. The participants were also assessed for their muscle spasms and the effects of the treatment on their breathing and movement. The researchers also used the Ashworth scales to measure their progress. The hydrotherapeutic intervention successfully reduced spasticity. There was a statistical improvement in Ashworth scores for both study and control groups (*p* < 0.01 and *p* < 0.02, respectively).

The participants in the hydrotherapy group exhibited a significant increase in their Functional Independence Measurement (FIM) scores. They also showed a decrease in oral baclofen consumption. Regarding spasm severity, the hydrotherapy group showed a significant decrease in spasm severity (*p* < 0.02). The control group’s spasm severity was 1.4, and the hydrotherapy group’s was 0.7 (respectively, *p* < 0.05, *p* < 0.001).

The researchers also noted that the use of hydrotherapy led to a drop in spasm severity. This is the only clinical trial that we found using our algorithm [1].

The second study selected was a 2017 systematic review made by Chunxiao Li et al. that evaluated the effects of aquatic exercise on the physical function and fitness of people with spinal cord injury. Four studies cited in the 2017 review showed that aquatic exercise combined with physiotherapy improved the participants’ functional independence. The programs were either a 10-week program or a 16-week program. A test–retest study conducted on eight participants revealed that underwater walking sessions, three times a week for eight weeks significantly improved their walking ability. A case study also showed that aquatic and land-based exercises can improve their physical fitness. The study showed that the 8-week underwater treadmill program led to a decrease in participants’ daily walking heart rate. A 15-week aquatic exercise program was found to improve participants’ swimming distance and force critical capacity. However, the program did not enhance the participants’ forced vital capacity (FVC) and forced expiratory flow rate. A similar study conducted on a 3-year program showed that combining swimming and physiotherapy sessions helped improve participants’ cardiorespiratory efficiency [1].

The third paper that met the criteria for selection in our review was a review published in 2018 by Terry J. et al. focused on the effects of aquatic exercise on patients with spinal cord injuries. The study analyzed the effects of different types of aquatic exercise on different aspects of health. It was theorized that the effects of buoyancy and hydrostatic pressure on the patients’ lumbopelvic hip complex and force closure improved their underwater walking. Additionally, the effects of buoyancy on the hip swing were proven to counteract the gravity-based effects of walking. The researchers also noted that the energy expenditure of patients decreased during underwater walking sessions [20,21]. The 1998 study conducted by Zamparo and Pagliaro from this 2018 [22] review was limited by its design and did not follow the usual follow-up procedures. As a result, it was not able to provide sufficient evidence supporting the study’s findings. While submerged in warm water, an exerciser’s heart rate lowers and enhances the thermoregulatory response of the body, which helps prolong the participant’s ability to exercise. This benefit is evidenced by the fact that water provides the ideal conductor of heat, which helps the body maintain a low core temperature. This allows the exerciser to endure longer and improve their energy-use efficiency. A study published in 2004 focused on the effects of hydrotherapy on SCI patients. The researchers discovered that a reduction in the dosage of the anti-inflammatory drug Baclofen significantly reduced muscle spasticity [22]. According to a 2009 study, the physiological effects of hydrotherapy on various conditions are still not clear. Further studies are needed to analyze the mechanism that influences this treatment’s effectiveness. The effects of hydrostatic pressure on the body are similar to those of Boyle’s law. Breathing underwater increases the body’s pressure, which makes breathing more costly. According to Becker, a patient’s vital capacity decreases by about 6% to 9% after performing underwater exercises due to hydrostatic pressure. This effect counters the inspiratory muscle action of underwater exercises. A study conducted in 1980 noted that SCI patients who participated in aquatic exercises improved their respiratory fitness. Other studies also suggest that performing aquatic exercises can improve a person’s expiratory muscle strength [22].

The fourth study we selected was a 2019 study made by Andresa R et al. that discussed the use of aquatic therapy by rehabilitation professionals for patients with spinal cord injuries. The researchers stated that aquatic therapy is a useful tool for improving the adherence of patients to their treatment. It does not require a standard technique or design [23].

**Table 1:** Appraisal of records according to the modified Downs and Black Appraisal Scale.

Downs and Black Appraisal						
Authors	Reporting (*n* = 5)	External Validity (*n* = 3)	Internal Validity (*n* = 3)	Power (*n* = 5)	Total (*n* =16)	Grading % = x/16 × 100
N. Kesiktas, N. Paker, N. Erdogan, G. Gülsen, D. Biçki, and H. Yilmaz (2004) [1]	5	3	2	4	14	87.5 % (very good)
C. Li, S. Khoo, A. Adnan (2017) [19]	5	3	3	4	15	93.75% (very good)
T. J. Ellapen, H. V. Hammill, M. Swanepoel, G. L. Strydom (2018) [20]	5	3	3	4	15	93.75% (very good)
A. R. Marinho-Buzelli, A. J. Zaluski, A. Mansfield, A. M. Bonnyman, K. E. Musselman (2019) [22]	5	1	3	3	11	68.75% (fair)

N, number; x, sum of Downs and Black appraisal.

Due to the low number of studies supporting the effectiveness of aquatic therapy for patients with spinal cord injuries, further studies are needed to establish a standard procedure for conducting follow-up studies.

In our research, we were able to use only four publications, assessed with Downs and Black Scale as shown in Table 1. Due to the low number of studies, there is need for further studies and standardized methodology to prove the benefits of hydrotherapy for SCI patients.

**Table 2:** Chronological overview of the characteristics and findings of the records (*n* = 4).

Characteristics of the Study				
**Authors**	**Type of Study**	Sample	Method	Findings
N. Kesiktas, N. Paker, N. Erdogan, G. Gülsen, D. Biçki, and H. Yilmaz (2004) [1]	Experimental non-randomised control	Hydrotherapy group: 10, mean age 32.13 ± 8.34, gender: 2 females and 8 males, injury time (months): 8.6 ± 5.5, FIM: 52 ± 14.13, Ashworth Score 3 ± 0.92, Oral Baclofen (mg) 96 ± 12, Etiology (accident) 50%. Control group: 10, mean age 33.10 ± 10.71, gender: 3 females and 7 males, injury time (months): 7.70 ± 6.06, FIM: 54.70 ± 18.8, Ashworth Score 2.50 ± 1.18, Oral Baclogen (mg) 100 ± 0, Etiology (accident) 50%.	The hydrotherapy group received 20 min of underwater exercises at 71 º F (21.6 º C) 3 times/week, and also participated in the usual rehabilitation, which included passive range of motion 2 times/day, psychotherapy and oral baclofen for 10 weeks. The control group were able to maintain their usual activities through the conventional rehabilitation program.	The hydrotherapeutic intervention successfully reduced spasticity and oral baclofen doses and raised FIM scores compared to control group.
C. Li, S. Khoo, A. Adnan (2017) [19]	Systematic review	A total of 143 participants with SCI were reported and the sample size of each study ranged from 1 to 60. More male participants were reported than female (male = 91, female = 52). Participants were adults aged between 18 and 63 years. A total of seven of eight studies reported participants’ injury levels on the spinal cord (the study by Pachalski and Mekarski did not report the specific injury level). Only 4 studies provided the grade of ASIA impairment scale. There was a big range in terms of postinjury time from 7 months to 28 years. In terms of study design, 3 were controlled clinical trials, 2 single group test–retest designs, 1 randomized controlled trial, 1 single-subject design, and 1 case study.	Eight of 276 studies met the inclusion criteria, of which none showed high research quality. Four studies assessed physical function outcomes and 4 studies evaluated aerobic fitness as outcome measures. Significant improvements on these 2 outcomes were generally found. Other physical or fitness outcomes including body composition, muscular strength, and balance were rarely reported.	There is insufficient evidence to support the efficacy of aquatic exercise on increasing physical function and aerobic fitness among SCI patients. We cannot yet draw any conclusion about the effectiveness of underwater training on body composition, muscular strength, and balance among the study population.
T. J. Ellapen, H. V. Hammill, M. Swanepoel, G. L. Strydom (2018) [20]	Systematic review	A total of 142 participants were reported (but 83 PWSCI), with sample sizes varying from 1 to 30 and participant age varying from 5 to 70 years. Five studies provided kinanthropometric characteristics, whereas 5 studies considered the number of years injured, and 10 studies described the aquatic exercise intervention. The overall quality of the studies was rated as fair (62.0%).	A literature surveillance was conducted between 1998 and 2017, through the Crossref meta-database and Google Scholar, according to the PRISMA procedures. Key search words were water-therapy, aquatic-therapy, hydrotherapy, spinal cord injury, rehabilitation, human, kinematics, underwater gait, cardiorespiratory, thermoregulation and spasticity. The quality of each paper was evaluated using a modified Downs and Black Appraisal Scale. The participants were recorded pertaining to SCI and hydrotherapy. The outcomes of interest were hydrotherapy interventions, the impact of hydrotherapy on gait kinematics, thermoregulation during water submersion, and cardiorespiratory function of PWSCI.	Hydrotherapy increases PWSCI underwater gait kinematics, cardiorespiratory and thermoregulatory responses and reduces spasticity.
A. R. Marinho-Buzelli, A. J. Zaluski, A. Mansfield, A. M. Bonnyman, K. E. Musselman (2019) [22]	Individual semi-structured interviews	None mentioned.	Six PT (2 male, 4 female), three PTA (female) and 1 KIN (female) participated. The following four themes were identified: (1) multi-system benefits from AT (e.g., from impairment to function, confidence, and enjoyment); (2) application of AT; (3) perceived barriers to implementing AT; and (4) water as an enabler to function on land. All were interviewed regarding their clinical findings while working with SCI patients in aquatic environment.	The participants reported AT was a unique and fickle approach that benefits the multi-dimensional aspects of the health of individuals with SCI/D. They inserted AT very well into their clinical practice despite the barriers professionals and clients face.

AT—Aqua therapy; PWSCI—Patient/SCI patients; SCI—Spinal cord injury; FIM—Functional independence measure; PTs—Physical therapists; PTA—PT assistants; KIN—kinesiologists; ASIA—American Spinal Cord Injury Association.

## 4. Discussion

The use of hydrotherapy in the treatment of the SCI patient decreases spasticity, increase underwater functioning of the lower limbs and gait kinematics, increase cardiorespiratory and thermoregulatory status, significant complications that can create a varying degree of disabilities. This therapy has a different mechanism of action on these complications through all the properties that therapeutic water can present: physical, chemical, and mechanical properties [1,20,24].

As it appears in the data from the specialized literature, spasticity can develop months or years after the acute spine cord injury and lead to severe function loss and hospitalization [25]. According to studies, 65–78% of the patients with chronic SCI (≥1 year postinjury) experience spasticity symptoms [26]. Up to 5 years after the spine cord injury, one-third of all patients have difficulties with spasticity [27]. Any medical intervention that leads to an improvement in neurological symptoms is vital for patient therapeutical management. Future clinical trials are needed to make significant arguments about the role of hydrotherapy.

Risk factors for cardiovascular diseases are increased in SCI patients [28]. SCI patients have considerably reduced daily energy expenditure due to a lack of motor function, as well as fewer opportunities to participate in physical exercise [29]. Additionally, SCI is associated with abnormal blood pressure, heart rate variability, arrhythmias, and a decreased cardiovascular response to exercise, which can restrict physical activity capacity [29]. Improving cardiorespiratory status through hydrotherapy is an important element for the SCI patient’s compliance in therapeutic management and the subsequent evolution of cardiac symptoms. Improving cardiovascular status through hydrotherapy is an important element for the SCI patient’s compliance with treatment and subsequent cardiac and respiratory symptoms evolution.

The possible effect of increasing underwater functioning of the lower limbs and gait kinematics is an important element that supports the need for future scientific studies on the role of hydrotherapy in the SCI patient.

Design and intervention methods are required for a successful study. They play an essential role in improving the quality of the study and its results.

A significant number of clinical case reports were discovered in the context of the research articles included. Although clinical case reports are not considered objective sources of information, they still play a vital role in the development and dissemination of medical knowledge.

The importance of clinical trials is often neglected. Although they may seem simple, they are conducted through a rigorous process that is governed by ethical principles.

Randomized controlled trials are often used to evaluate the effectiveness of interventions and have been acknowledged as the gold standard because they are considered one of the most powerful types of evidence for use in the development of evidence-based clinical therapeutical programs [30]. However, randomized controlled trials have often been poorly reported in medical journals [31,32].

It is essential to find solutions for implementing randomized controlled trials in medical rehabilitation units, to support the use and effectiveness of balneal factors in the treatment of various orthopedic and neurologic diseases [32], and many literature data with different studies in various pathology can be used as model [33].

The fact that when the PRISMA algorithm was applied the number of articles that could be included in the study in the current review was minimal compared to the vast number of patients with neurological pathology studied is of importance in medical rehabilitation as well as a treatment methodology, and arguments were sought for this aspect. One of the hypotheses is that there is an actual development of other treatments, such as robotic, computerized, or immersion in virtual reality.

The exoskeleton systems represent a new therapeutic approach in the rehabilitation program of these patients and can be successfully used for gait re-education, proposing kinetic models of the exoskeleton with permanent control over the angle, and angular velocity at the level of each joint, as well as over the parameter muscles (muscle parameters), offering the patient continuous passive movement [34,35]. This new therapy, along with the safety offered to the patient during treatment, reduces the therapist’s workload and therapy cost [34]. The cost level of a rehabilitation program will always represent a significant concern of any health service so that as many patients as possible can benefit from rehabilitation services. The exoskeleton has much potential to maintain good health and improve the QOL in individuals with SCI. Of course, future studies are needed in this therapeutic sector as well.

Another therapeutic class used in the treatment of spasticity in patients with spinal cord injury in current medical rehabilitation is botulinum toxin. There are standardized schemes that can also be customized for the individual patient’s treatment situation and the patient’s target muscles, and the treatment is more effective in the first 6 months [36,37,38].

The strength of the study is as follows: The number of patients with neurological pathology in the field of spinal cord injury is very high, and the disability resulting from this pathology has a significant impact on the quality of life of both the patient and his family. For this reason, it is necessary to investigate all the therapeutic possibilities used to increase independence and maintain the existing functional remainder. Hydrokinetotherapy is part of the therapeutic rehabilitation management of these pathologies, with a therapeutic impact on several systems, as discussed in the Section 1. The study was based on the PRISMA research algorithm necessary for conducting systematic reviews. The period included in the research is essential, namely 2000–2021. The databases included in the research have medical scientific value, with broad international recognition: PubMed, PubMed Central, Science Direct, Scopus, and SpringerLink. The subject is of interest both for the medical rehabilitation specialty and for neurological specialists because the therapy studied with an impact on the symptomatology that induces a high degree of disability can lead to a decrease in the doses of medication with a neurological impact, which increases the patient’s compliance.

The limitations of the study are as follows: The small number of studies in specialized literature regarding the impact of hydrokinetotherapy on patients with spinal cord injury makes it impossible to carry out a meta-analysis on this subject and very difficult to carry out a review that respects all the stages imposed by the PRISMA algorithm. The fact that the review was not pre-registered in an international prospective register of systematic reviews, such as PROSPERO, and a funnel plot was not made are also a limitation of this review. In addition to the limited number of existing scientific studies, there are other drawbacks, such as heterogeneous methodologies, disparate study populations, and different training programs, which must be overcome in future studies.

## 5. Conclusions

Due to the low quality of the controlled studies and the lack of sufficient follow-up randomized controlled trials, there is not enough evidence supporting the importance of hydrotherapy for the functional rehabilitation of patients with spinal cord injury. Hydrotherapy’s physiologic effects in orthopedic recovery and neurological disease must be investigated further. Financial support for such studies and the required clinical trials are still an issue. Despite its significant therapeutic benefits, aquatic exercise and balneal rehabilitation are underutilized.

Rehabilitation techniques must represent the main purpose of multiple and important qualitative scientific studies because medical rehabilitation represents a key role in spinal disorders such as SCI.

## References

[1] Kesiktas N., Paker N., Erdogan N., Gülsen G., Biçki D., Yilmaz H.G. (2004). The use of hydrotherapy for the management of spasticity. Neurorehabil. Neural Repair.

[2] Priebe M.M., Sherwood A.M., Thornby J.I., Kharas N.F., Markowski J. (1996). Clinical assessment of spasticity in spinal cord injury: A multidimensional problem. Arch. Phys. Med. Rehabil..

[3] Hippocrates On Airs, Waters, and Places. 400 BC. http://classics.mit.edu/Hippocrates/airwatpl.html.

[4] Kneipp S. (1894). My Water Cure, as Tested through more than Thirty Years, and Described for the Healing of Diseases and the Preservation of Health.

[5] Hall J., Swinkels A., Briddon J., McCabe C.S. (2008). Does aquatic exercisere lieve pain in adults with neurologic or musculoskeletal disease? A systematic reviewand meta-analysis of randomized controlled trials. ArchPhys. Med. Rehabil..

[6] Bender T., Karaglle Z., Balint G.P., Gutenbrunner C., Balint P.V., Sukenik S. (2005). Hydrotherapy, balneotherapy, and spa treatment in pain management. Rheumatol. Int..

[7] Yamazaki F., Endo Y., Torii S., Sagawa S., Shiraki K. (2000). Continuous monitoring of change in hemodilution during water immersion in humans: Effect of water temperature. Aviat. Space Environ. Med..

[8] Gabrielsen A., Ek V.A., Johansen L.B., Warberg J., Christensen N.J., Pump B., Norsk P. (2000). Forearm vascular and neuroendocrine responses to graded water immersion in humans. Acta PhysioScand.

[9] Fam A.G. (1991). Spa treatment in arthritis: A rheumarologist’s view. J. Rheumatol..

[10] Giesecke C., Ruoti R.G., Morris D.M., Cole A.J. (1997). Aquatic rehabilitation of clients with spinal cord injury. Aquatic Rehabilitation.

[11] Stiens S.A., Biener-Bergman S., Goetz L.L. (1997). Neurogenic boweldys function after spinal cord injury: Clinical evaluation and rehabilitation management. ArchPhys. Med. Rehab..

[12] Recio A.C., Cabahug P. Safety of aquatic therapy for adults with complex medical conditions among chronic spinal cord injury. Proceedings of the ASCIP Annual Meeting.

[13] Stiens S.A., Shamberg S., Shamberg A., Guistini A., O’Young B.J., Young M.A., Stiens S.A. (2007). Environ mental barriers: Solutions to participation, collaboration and to getherness. Physical Medicine and Rehabilitation Secrets.

[14] Stiens S.A., O’Young B.J., Young M.A., O’Young B.J., Young M.A., Stiens S.A. (2007). Person-centered rehabilitation: Interdisciplinary intervention to enhance patient enablement, physical medicine and rehabilitation secrets. Physical Medicine and Rehabilitation Secrets.

[15] Liberati A., Altman D.G., Tetzlaff J., Mulrow C., Gøtzsche P.C., Ioannidis J.P.A., Clarke M., Devereaux P.J., Kleijnen J., Moher D. (2009). The PRISMA Statement for Reporting Systematic Reviews and Meta-Analyses of Studies That Evaluate Health Care Interventions: Explanation and Elaboration. PLoS Med..

[16] Downs S.H., Black N. (1998). The feasibility of creating a checklist for the assessment of the methodological quality both of randomized and nonrandomized studies of health care interventions. J. Epidemiol. Community Health.

[17] Machado M., Bajcar J., Guzzo G.C., Einarson T.R. (2007). Sensitivity of patient out comes to pharmacist interventions. Part II: Systematic reviewand meta-analysis in hypertension management. Ann. Pharmacother..

[18] Kamioka H., Tsutani K., Okuizumi H., Mutoh Y., Ohta M., Handa S., Okada S., Kitayuguchi J., Kamada M., Shiozawa N. (2011). A systematic review of nonrandomized controlled trials on the curative effects of aquatic exercise. Int. J. Gen. Med..

[19] Li C., Khoo S., Adnan A. (2017). Effects of aquatic exercise on physical function and fitness among people with spinal cord injury. Medicine.

[20] Ellapen T.J., Hammill H.V., Swanepoel M., Strydom G.L. (2018). Strydom, The benefits of hydrotherapy to patients with spinal cord injuries. Afr. J. Disabil..

[21] Zamparo P., Pagliaro P. (2007). The energy cost of level walking before and after hydro-kinesi therapy in patients with spastic paresis. Scand. Med. Sci. Sport.

[22] Marinho-Buzelli A.R., Zaluski A.J., Mansfield A., Bonnyman A.M., Musselman K.E. (2019). The use of aquatic therapy among rehabilitation professionals for individuals with spinal cord injury or disorde. J. Spinal. Cord. Med..

[23] Wall T., Falvo L., Kesten A. (2017). Activity-specific aquatic therapy targeting gait for a patient with incomplete spinal cord injury. Physiother. Theory Pract..

[24] Munteanu C. (2013). Therapeutic Mineral Waters.

[25] Rekand T., Hagen E., Grønning M. (2012). Marit Grønning, Spasticity following spinal cord injury. Tidsskr. Nor. Legeforen..

[26] Adams M.M., Hicks A.L. (2005). Spasticity after Spinal Cord Injury. Spinal Cord.

[27] Holtz K.A., Lipson R., Noonan V.K., Kwon B.K., Mills P.B. (2017). The prevalence and Effect of Problematic Spasticity After Traumatic Spinal Cord Injury. Arch. Phys. Med. Rehabil..

[28] Cragg J.J., Noonan V.K., Krassioukov A., Borisoff J. (2013). Cardiovascular disease and spinal cord injury. Neurology.

[29] Myers J., Lee M., Kiratli J. (2007). Cardiovascular Disease in Spinal Cord Injury An Overview of Prevalence, Risk, Evaluation, and Management. Am. J. Phys. Med. Rehabil..

[30] Moher D., Dulberg C., Wells G. (1994). Statistical power, sample size and their reporting in randomized controlled trials. J. Am. Med. Assoc..

[31] Schulz K. (1996). Randomized trials, human nature, and reporting guidelines. Lancet.

[32] Day S., Altman D. (2000). Blinding to clinical trials and other studies. Br. Med. J..

[33] Gasmi A., Bjorklund G., Mujawdiya P.K., Semenova Y., Peana M., Dosa A., Piscopo S., Benahmed A.G., Costea D.O. (2021). Micronutrients deficiencies in patients after bariatric surgery. Eur. J. Nutr..

[34] Głowiński S., Ptak M. (2022). A kinematic model of a humanoid lower limb exoskeleton with pneumatic actuators. Acta Bioeng. Biomech..

[35] Pons J.L. (2008). Wearable Robots.

[36] Stevenson V.L. (2010). Rehabilitation in practice: Spasticity management. Clin. Rehabil..

[37] Little J.W., Micklesen P., Umlauf R., Britell C. (1989). Lower extremitiy manifestations in spasticity in chronic spinal cord injury. Am. J. Phys. Med. Rehabil..

[38] Horga Parte J.F., Pareés M.I., López d.V.L.J., Castor G.A. (2010). Toxina botulínica: Origen, estructura, actividad farmacológica y cinética. Toxina Botulínica.

